# The methyl jasmonate-responsive transcription factor SmMYB1 promotes phenolic acid biosynthesis in *Salvia miltiorrhiza*

**DOI:** 10.1038/s41438-020-00443-5

**Published:** 2021-01-01

**Authors:** Wei Zhou, Min Shi, Changping Deng, Sunjie Lu, Fenfen Huang, Yao Wang, Guoyin Kai

**Affiliations:** 1 Laboratory of Medicinal Plant Biotechnology, College of Pharmacy, Zhejiang Chinese Medical University, 310053 Hangzhou, Zhejiang China; 2 Institute of Plant Biotechnology, School of Life Sciences, Shanghai Normal University, 200234 Shanghai, China

**Keywords:** Secondary metabolism, Transcription factors

## Abstract

Water-soluble phenolic acids are major bioactive compounds in the medicinal plant species *Salvia miltiorrhiza*. Phenolic acid biosynthesis is induced by methyl jasmonate (MeJA) in this important Chinese herb. Here, we investigated the mechanism underlying this induction by analyzing a transcriptome library of *S. miltiorrhiza* in response to MeJA. Global transcriptome analysis identified the MeJA-responsive R2R3-MYB transcription factor-encoding gene *SmMYB1*. Overexpressing *SmMYB1* significantly promoted phenolic acid accumulation and upregulated the expression of genes encoding key enzymes in the phenolic acid biosynthesis pathway, including cytochrome P450-dependent monooxygenase (CYP98A14). Dual-luciferase (dual-LUC) assays and/or an electrophoretic mobility shift assays (EMSAs) indicated that SmMYB1 activated the expression of *CYP98A14*, as well as the expression of genes encoding anthocyanin biosynthesis pathway enzymes, including chalcone isomerase (CHI) and anthocyanidin synthase (ANS). In addition, SmMYB1 was shown to interact with SmMYC2 to additively promote *CYP98A14* expression compared to the action of SmMYB1 alone. Taken together, these results demonstrate that SmMYB1 is an activator that improves the accumulation of phenolic acids and anthocyanins in *S. miltiorrhiza*. These findings lay the foundation for in-depth studies of the molecular mechanism underlying MeJA-mediated phenolic acid biosynthesis and for the metabolic engineering of bioactive ingredients in *S. miltiorrhiza*.

## Introduction

*Salvia miltiorrhiza* Bunge (Lamiaceae family) is a valuable traditional Chinese medicinal plant species whose common name is Danshen^1,2^. Dried roots and rhizomes of Danshen have historically been used to treat cardiovascular and cerebrovascular diseases, and they are reported to have pharmacological activities^3–5^. Several Danshen products, including Fufang Danshen dripping pills, are used widely in clinical practice^6,7^. The bioactive ingredients in *S. miltiorrhiza* include water-soluble phenolic acids such as salvianolic acid A (Sal A), salvianolic acid B (Sal B), caffeic acid (CA), and rosmarinic acid (RA)^8–12^. In commercial Danshen decoctions, Sal B is the major marker component used for quality control according to the official Chinese Pharmacopoeia^13,14^. These compounds have attracted increased amounts of attention in recent years.

**Fig. 1:**
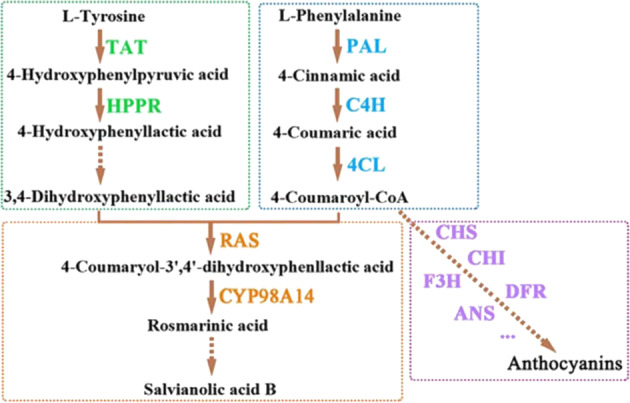
Phenolic acid biosynthesis pathway and branched anthocyanin biosynthesis pathway. The green dotted frame indicates the tyrosine-derived pathway, the blue dotted frame indicates the phenylpropanoid pathway, the orange dotted frame indicates the downstream biosynthesis pathway of phenolic acids, and the purple dotted frame indicates the anthocyanin biosynthesis pathway

Given the widespread use of Danshen, it is important to improve the production of phenolic compounds in *S. miltiorrhiza* to meet clinical demands. Phenolic acid biosynthesis in *S. miltiorrhiza* involves two parallel pathways: the general phenylpropanoid pathway and the tyrosine-derived pathway^13,15^. In the phenylpropanoid pathway, l-phenylalanine is metabolized to 4-coumaroyl-CoA via phenylalanine ammonia-lyase (PAL), cinnamic acid 4-hydroxylase (C4H), and 4-coumarate-CoA ligase (4CL)^16,17^. In the tyrosine-derived pathway, 3,4-dihydroxyphenyllactic acid (DHPL) is generated from l-tyrosine via tyrosine aminotransferase (TAT) and 4-hydroxyphenylpyruvate reductase (HPPR), as well as additional unresolved catalytic steps, to form 3,4-dihydroxyphenyllactic acid^7^. These two products are subsequently catalyzed by rosmarinic acid synthase (RAS) and a cytochrome P450-dependent monooxygenase (CYP98A14) to form RA^15^. Other detailed steps in these pathways have not yet been characterized, and the enzymes used for the biosynthesis of water-soluble phenolic acid medicinal substances such as Sal B have not yet been identified^7,18,19^. 4-Coumaroyl-CoA in the phenylpropanoid pathway of phenolic acid biosynthesis is also the primary precursor for anthocyanin biosynthesis (Fig. 1). Important catalytic enzymes in the anthocyanin biosynthesis pathway include chalcone synthase (CHS), chalcone isomerase (CHI), flavone 3-hydroxylase (F3H), dihydroflavonol 4-reductase (DFR), and anthocyanidin synthase (ANS)^13,20,21^.

Metabolic engineering is an efficient approach for improving the production of active ingredients in medicinal plants. This process requires a deep understanding of the biosynthesis pathways of active ingredients, including both biosynthesis-related and regulatory genes^22–25^. Although great progress has been made in the isolation and functional identification of biosynthesis-related genes involved in medicinal compounds, little is known about the transcriptional regulation of phenolic acid biosynthesis in *S. miltiorrhiza*. In addition, phenolic acid biosynthesis is induced by MeJA treatment^18,19^, but the underlying regulatory mechanism is not clear. To determine how MeJA regulates phenolic acid biosynthesis, we previously performed a transcriptome analysis of hairy roots of *S. miltiorrhiza* under MeJA treatment and demonstrated that MYB transcription factor (TF) genes are differentially expressed in response to MeJA^19^, which implied that MYB TFs may be involved in regulating the biosynthesis of phenolic acids in *S. miltiorrhiza*.

MYB TFs are classified into four subfamilies based on the number of repeats of the conserved MYB domain (involved in DNA binding) in their N-terminal regions: 1R-MYBs, R2R3-MYBs, R1R2R3-MYBs, and 4R-MYBs. R2R3-MYBs (two-repeat MYBs) are the most common type of MYB TF in plants^26–28^ and have many roles in regulating plant development, growth, defense, secondary metabolism, and other biological functions^28^. For example, the R2R3-MYB TF EsMYBA1 can activate the promoters of dihydroflavonol 4-reductase (DFR) and anthocyanidin synthase (ANS) genes to promote anthocyanin accumulation in the medicinal plant species *Epimedium sagittatum*^29^. The apple MYB TF *MdMYB3* has been reported to regulate anthocyanin biosynthesis and flower development^30^. Moreover, in *S. miltiorrhiza*, overexpressing *SmMYB36* in hairy roots significantly decreased the expression of most genes of the phenylpropanoid pathway (such as *PAL1* and *C4H1*) and the tyrosine pathway (such as *TAT1* and *HPPR1*), thereby inhibiting phenolic acid accumulation^31^. *SmMYB39* negatively regulates the transcription and enzyme activities of C4H and TAT, thus decreasing rosmarinic acid contents, as demonstrated in transgenic hairy roots overexpressing *SmMYB39*^32^. However, very few R2R3-MYB TFs that act as positive regulators in promoting phenolic acid accumulation have been identified, and the molecular mechanism regulating phenolic acid biosynthesis induced by MeJA in *S. miltiorrhiza* remains unknown.

Since MYB TFs have important roles in plant secondary metabolism and have a positive effect on MeJA-inducible phenolic acid biosynthesis, it is important to investigate the roles of MYB TFs in regulating phenolic acid biosynthesis in *S. miltiorrhiza*. In the current study, we identified the MeJA-responsive R2R3-MYB transcription factor-encoding gene *SmMYB1* from an *S. miltiorrhiza* MeJA-induced transcriptome library and demonstrated that SmMYB1 activated *CYP98A14*, *CHI*, and *ANS* expression and separately promoted the accumulation of both phenolic acids and anthocyanins. Moreover, SmMYB1 was shown to interact with SmMYC2 to form an MYB1/MYC2 complex that additively promotes the expression of *CYP98A14* compared to the action of SmMYB1 alone. Our findings thus provide insights into the molecular mechanisms underlying MeJA-induced phenolic acid and anthocyanin biosynthesis in *S. miltiorrhiza*.

## Materials and methods

### Plant materials and elicitor preparation

*S. miltiorrhiza* plants were grown in a greenhouse at Zhejiang Chinese Medical University or cultivated on Murashige and Skoog (MS) media at 25 °C under a 16 h light/8 h dark photoperiod^4,33^. *Nicotiana benthamiana* seeds were sown in pots under the same conditions as those of *S. miltiorrhiza*^4,34^. Different tissues from a single one-year-old *S. miltiorrhiza* plant, including root, stem, leaf, petiole, calyx, and petal tissues, were collected for RNA isolation. The elicitors methyl jasmonate (MeJA), abscisic acid (ABA), gibberellic acid 3 (GA_3_), yeast extract (YE), salicylic acid (SA), and ethylene (Eth) were prepared as described by Zhang et al.^34^. Authentic tanshinone and phenolic acid standards (Aladdin, China) were prepared according to the methods of Shi et al.^4^.

### Isolation and characterization of *SmMYB1*

The open reading frame (ORF) of *SmMYB1* was cloned as described by Zhou et al.^35^. The primer pair used for gene cloning is listed in Supplementary Table S1. SmMYB1 was subjected to BLAST-Protein (BLASTP) analysis via the nonredundant (NR) protein sequence database (www.ncbi.nlm.nih.gov). *Scutellaria taiwanensis* StMYB13 (accession number AKA59794.1), *Scutellaria indica* SiMYB13 (AKA59777.1), and *Sesamum indicum* SiMYB5 (XP_011070362.1), which are highly homologous to SmMYB1 (MN400427), were subjected to amino acid sequence alignment using Vector NTI software (Invitrogen, USA). Sequence alignment and phylogenetic analysis were performed using ClustalX^36^. A phylogenetic tree was constructed based on amino acid sequences by the neighbor-joining method using MEGA 6.0 software, and the reliability of each node in the tree was evaluated using the bootstrap method, with 1000 replicates^37^.

### Quantitative real-time PCR (qRT-PCR)

The qRT-PCR analysis was performed as described by Shi et al.^38^. Different tissues (root, stem, leaf, petiole, calyx, and petal tissues) were collected from a single one-year-old plant and from hairy roots treated with MeJA, abscisic acid (ABA), gibberellin (GA_3_), yeast extract (YE), salicylic acid (SA) and ethylene (Eth), after which the tissue samples were frozen in liquid nitrogen. Total RNA was extracted as described previously^34^. A qRT-PCR assay was performed using a GoTaq-qPCR Master Mix kit (Promega, China) on an Applied Biosystems StepOne Real-time PCR System (USA). The internal control was the *S. miltiorrhiza Actin* gene. All the primers used for qRT-PCR are listed in Supplementary Table S1. Gene expression levels were quantified using the comparative *Ct* method^4,5^.

### Subcellular localization of SmMYB1

The full-length ORF of *SmMYB1* (without the stop codon) was fused to a green fluorescent protein (GFP) gene under the control of the CaMV 35S promoter within a *pMON530* vector to generate a *SmMYB*-GFP fusion protein as described previously^4,5^ (Supplementary Fig. S1). The constructs were subsequently transferred into *Agrobacterium tumefaciens* GV3101, which were then infiltrated into five-week-old *N. benthamiana* leaves for transient transformation, as described previously^5^. *pMON530*-GFP was used as a control. To visualize the nuclei, 4′,6-diamidino-2-phenylindole dihydrochloride (DAPI) solution (10 μg/mL) was injected into *N. benthamiana* leaves with a syringe 3 h prior to microscopy observations. YFP and DAPI signals were excited at 488 and 405 nm, respectively. At 48 h after infiltration, the GFP signals were visualized using a confocal spectral microscope imaging system (Leica TCS SP5).

### Generating *S. miltiorrhiza* hairy roots

The complete ORF of *SmMYB1* was cloned into the *Spe*I and *Bst*EII restriction enzyme sites of *pCAMBIA2300*^+^ under the control of the CaMV 35S promoter and NOS terminator to generate a *pCAMBIA2300*^+^–*SmMYB1* construct (Supplementary Fig. S2). The DNA sequence encoding SRDX (LDLDLELRLGFA) was inserted into the ORF of *SmMYB1* before the stop codon (TAA)^26^. This sequence was then subcloned into the *Spe*I and *Bst*EII sites of a *pCAMBIA2300*^+^ vector to generate a *pCAMBIA2300*^+^-*SmMYB1*-*SRDX* construct (Supplementary Fig. S2). All the constructs were introduced into *Agrobacterium* strain C58C1, which were then transformed into *S. miltiorrhiza* to produce transgenic lines with hairy roots, as described previously^5,38^.

### Measuring phenolic acid and anthocyanin contents

Hairy roots grown in shaker flasks were harvested and dried in an oven. The dried roots were ground to a powder, after which 200 mg samples were extracted with 16 mL of methanol/dichloromethane (3:1, v/v), sonicated for 1 h, and centrifuged at room temperature. Phenolic acid was extracted from the samples for HPLC analysis, as described previously^5,12,14^. The phenolic acids Sal A, Sal B, CA, and RA were extracted from the selected transgenic lines and quantified by comparison with standard curves and the corresponding retention times. Total phenolic acids were designated as TPA. Anthocyanin contents were determined by spectrophotometry by measuring the absorbance at 530 nm and 657 nm, with solutions without extracts used as blank controls^39^.

### Purification of recombinant proteins

cDNA from *SmMYB1* was amplified by PCR and cloned into a pETMAL_C_-H vector (Supplementary Fig. S3), which was then transformed into *Escherichia coli* BL21 cells. All the primers used are listed in Supplementary Table S1. SmMYB1 proteins were expressed in *E. coli* via induction with 0.5 mM isopropyl b-d-1-thiogalactopyranoside (IPTG), followed by incubation at 37 °C for 3 h and purification using amylose resin (Shanghai Sangon Biotech Co., Ltd.).

### Electrophoretic mobility shift assays (EMSAs)

EMSAs were performed using a Light Shift Chemiluminescent EMSA Kit (Thermo, USA) according to the manufacturer’s instructions, with minor modifications. Briefly, ~2 mg of the purified fusion protein was inputted into the binding reaction^12^, which was performed at 25 °C for 15 min in a thermal cycler (Bio-Rad). Biotin-labeled probes were synthesized by Shanghai Sangon Biotech Co., Ltd.; the sequences of the probes are listed in Supplementary Table S1.

### Dual-luciferase (dual-LUC) assays

Dual-LUC assays were performed as reported previously^14^. The primer pairs used to clone the promoters of genes involved in phenolic acid biosynthesis are listed in Supplementary Table S1. The promoter regions of candidate genes (~2000 bp) were inserted into A pGreen 0800-LUC vector driving a firefly LUC reporter gene, and the *Renilla* luciferase (REN) gene was controlled by the 35S promoter (Fig. S4). The recombinant vectors were subsequently transferred into *A. tumefaciens* (GV3101) together with a helper plasmid (pSoup-P19) encoding a cosuppressor. *N. benthamiana* leaves were then transiently transformed with the constructs by infiltrating the *Agrobacterium* mixture into the abaxial sides of the leaves. The infected area was harvested, extracts were evaluated using a Dual-Luciferase Reporter Assay System (Promega, Madison, USA), and, after 48 h, the fluorescence values of LUC and REN were detected with a luminometer. The LUC/REN ratio indicated the ability of SmMYB1 to bind to and activate gene promoters.

### Yeast two-hybrid (Y2H) assays

A Y2H assay was conducted using the Matchmaker Gold Yeast Two-Hybrid System according to the manufacturer’s instructions (Takara, Japan). The complete ORFs of *SmMYB1* and *SmMYC2* were amplified by PCR and cloned into the pGADT7 and pGKBT7 vectors, respectively, using the primers listed in Supplementary Table S1. The recombinant vectors were subsequently transformed into yeast strain AH109, and the autoactivation of pGKBT7-*SmMYB1* was tested.

The bait and prey constructs were cotransformed into AH109 yeast cells and grown on double dropout (DDO) media for three days. The transformed colonies were then plated onto selection media to assess the possible interactions between proteins (Clontech, USA).

### Bimolecular fluorescence complementation (BiFC) assays

To conduct BiFC assays, the ORF of *SmMYB1* fused to *cYFP* and the ORF of *SmMYC2* fused to *nYFP* were cloned into pXY104 and pXY106 vectors, respectively (Supplementary Fig. S5). The resulting constructs were introduced into *A. tumefaciens* strain GV3101, which were then infiltrated into 6-week-old *N. benthamiana* leaves using a 1 mL needleless syringe, as described previously^40^. The infected leaves were examined 48 h after infiltration. YFP fluorescent signals were captured using a confocal spectral microscopy imaging system (Leica TCS, SP5).

### Statistical analysis

All experiments performed in this study, including those involving PCR identification, qRT-PCR, HPLC, dual-LUC assays, and EMSAs, were repeated three times. Gene expression levels, phenolic acid contents, and anthocyanin contents were expressed as the means ± standard deviations (SDs). SPSS 11.5 software (SPSS) was used to analyze statistical significance by single-sample *t*-tests and one-way analysis of variance.

## Results

### Isolation and characterization of *SmMYB1*

We used a previously established MeJA-treated Danshen hairy root transcriptome database (0 h, untreated control; treated for 1 and 6 h with MeJA) (available in the Sequence Read Archive [SRA] of the National Center for Biotechnology Information [NCBI] public database under accession number GSE100970) to identify R2R3-MYB TFs related to phenolic acid biosynthesis in *S. miltiorrhiza*^19^. The expression levels of forty-two R2R3-MYB TF-encoding genes in the transcriptome database were upregulated in response to MeJA treatment (RPKM fold change > 2, Supplementary Fig. S6). Among these, we selected the MYB TF-encoding gene *SmMYB1* (*SmiContig1043*, GenBank accession number MN400427), whose expression profile was similar to that of the phenolic acid biosynthesis pathway genes *TAT1*, *PAL1*, *HPPR1*, *RAS1*, and *CYP98A14*, for functional analysis (Fig. S6).

**Fig. 2:**
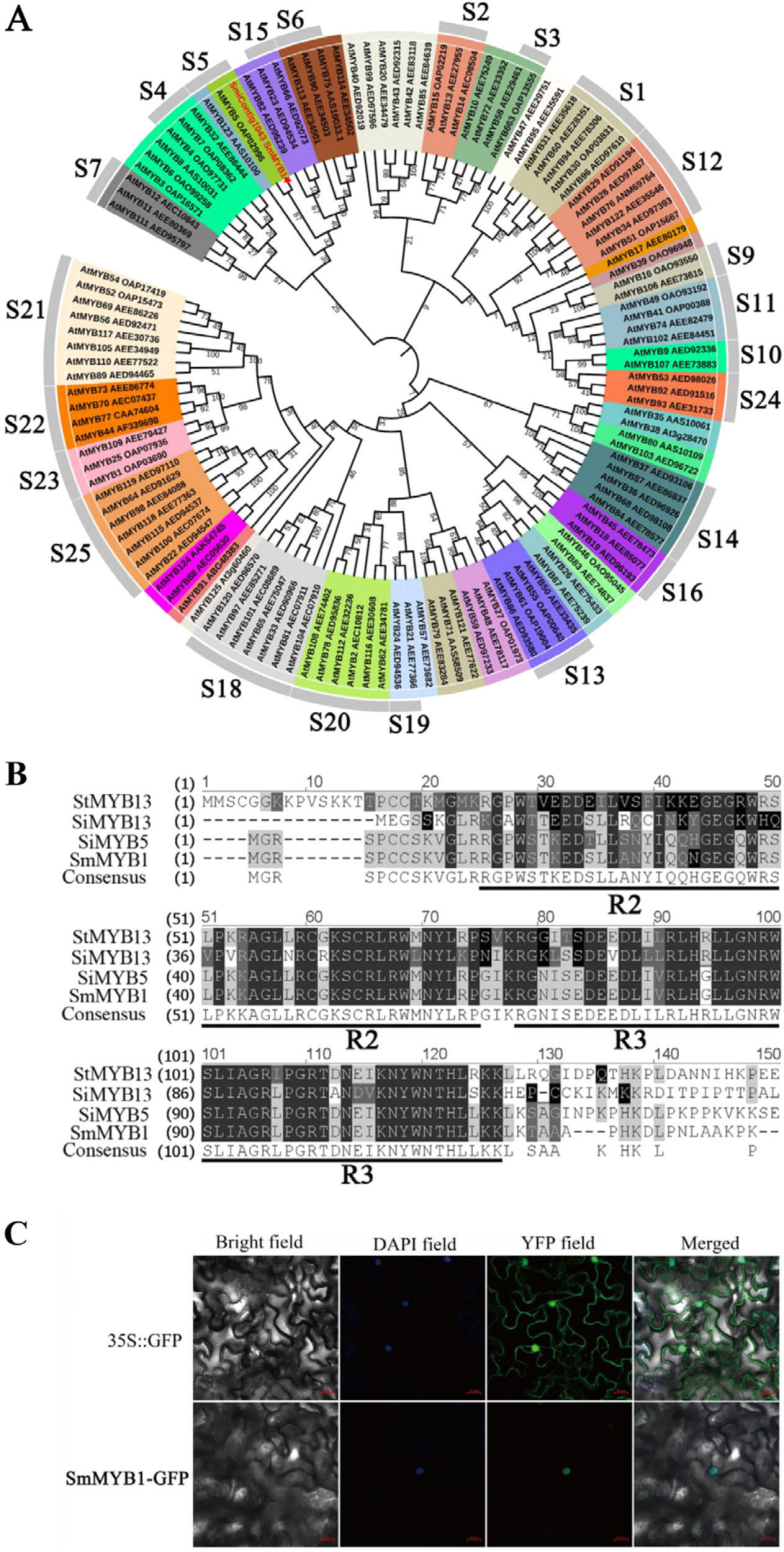
Characterization and subcellular localization of SmMYB1. **A** Phylogenetic tree comprising SmMYB1 in *S. miltiorrhiza* and 125 R2R3-MYB TFs in *A. thaliana* generated by the neighbor-joining method using MEGA 6.05 software. The numbers on the nodes indicate the bootstrap values after 1000 replicates. **B** Multiple amino acid sequence alignment of R2R2-MYB TFs. The underlines represent conserved domains. **C** Subcellular localization of SmMYB1 in *N. benthamiana* leaf epidermal cells. DAPI was used as a positive control

The full-length cDNA sequence of *SmMYB1* contained an ORF of 645 bp. We constructed a phylogenetic tree comprising SmMYB1 and 125 R2R3-MYB TFs in *A. thaliana* (Fig. 2A). BLAST-Protein (BLASTP) analysis showed that SmMYB1 shared the highest homology (85.6%) with *Sesamum indicum* MYB5 (SiMYB5); both proteins contained two conserved R2 and R3 repeats at the N-terminus, as revealed by amino acid sequence alignment (Fig. 2B). SmMYB1 shares the closest evolutionary relationships with members of the S5 subfamily (AtMYB5 and AtMYB123), S15 subfamily (AtMYB23 and AtMYB66), S6 subfamily (AtMYB75, AtMYB90, AtMYB113, and AtMYB114), S4 subfamily (AtMYB4, AtMYB7, and AtMYB32), and S7 subfamily (AtMYB11, AtMYB12, and AtMYB111).

### Expression analysis of *SmMYB1*

**Fig. 3:**
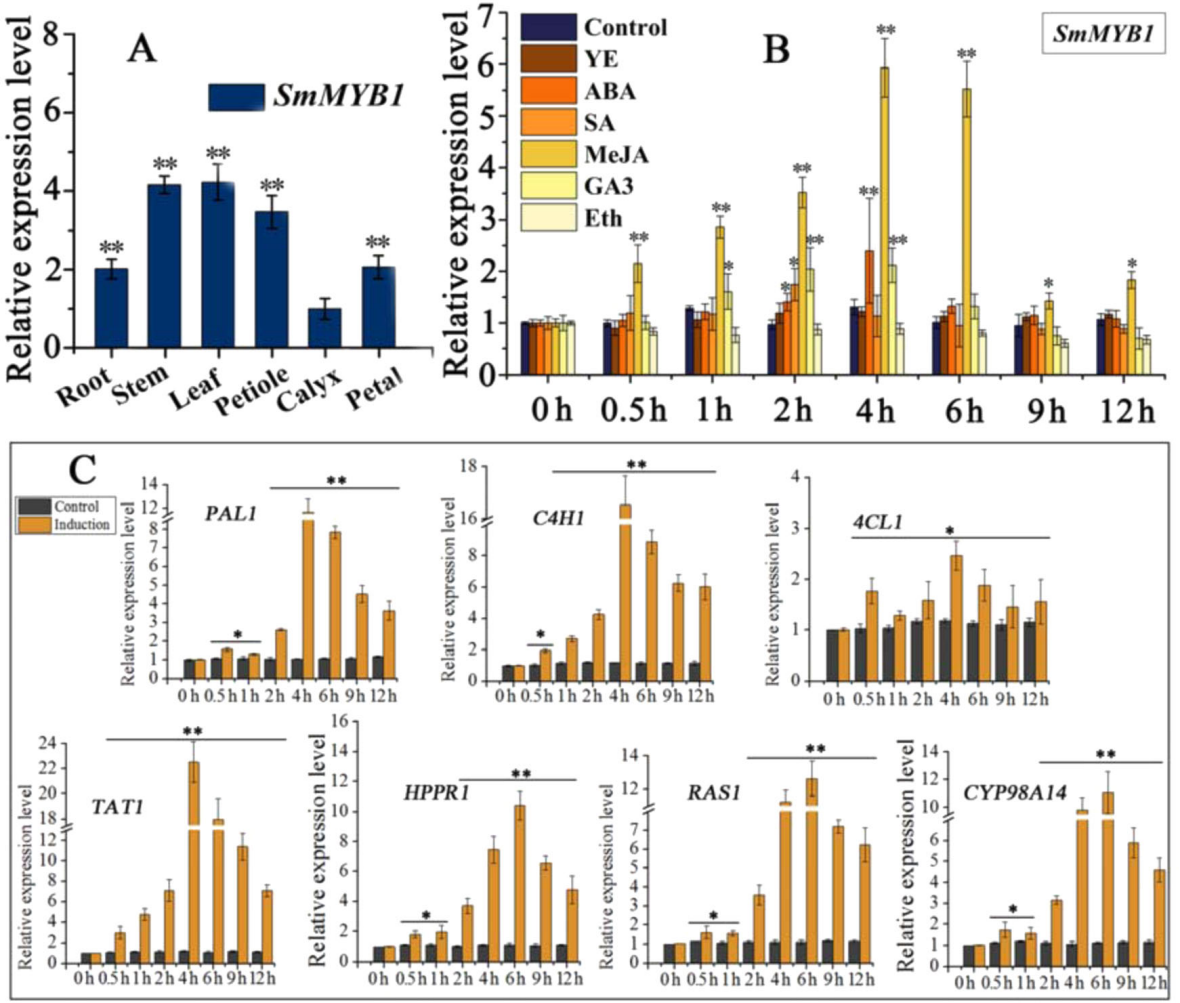
Expression profiles of *SmMYB1* and genes involved in the phenolic acid biosynthesis pathway in response to treatment with MeJA. **A** Expression patterns of *SmMYB1* in different tissues. The fold changes of the relative gene expression level in all the other tissues are normalized to the expression level in the calyx tissue. **B** Effect of elicitors (yeast extract, YE; abscisic acid, ABA; salicylic acid, SA; methyl jasmonate, MeJA; gibberellic acid, GA_3_; ethylene, Eth) on *SmMYB1* expression. **C** Expression profiles of genes in the phenolic acid biosynthesis pathway after treatment with MeJA. The fold changes in the relative gene expression level were all normalized to the control expression without induction at the 0 h time point in **B** and **C**. The error bars indicate the standard errors of the means. The asterisks indicate significant differences between the calyx versus the other tissues in **A**, between the treatment versus the control in **B**, and according to **C** *t-*tests at two different significance levels (**P < 0.01; *P < 0.05). The experiment was replicated three times

*SmMYB1* is expressed in the nucleus, as revealed by transient expression analysis of epidermal cells from young *N. benthamiana* leaves (Fig. 2C). We examined the expression profiles of *SmMYB1* in different tissues of *S. miltiorrhiza* by qRT-PCR. *SmMYB1* was expressed ubiquitously in all tissues examined, including root, stem, leaf, petiole, calyx, and petal tissues, with the highest expression in the leaf tissue and the lowest in the calyx tissue (Fig. 3A). We also examined the expression of *SmMYB1* in *S. miltiorrhiza* hairy roots after treatment with YE, ABA, SA, MeJA, GA_3_, and Eth. *SmMYB1* expression was significantly upregulated in response to MeJA, with expression peaking after 4 h of MeJA treatment to levels 4.5-fold higher than those of the control (Fig. 3B). Seven genes for phenolic acid biosynthesis, including *PAL1*, *TAT1*, *RAS1*, and *CYP98A14*, showed various degrees of upregulation after MeJA treatment (Fig. 3C). These results indicate that *SmMYB1* is induced in response to MeJA treatment and is expressed at the highest levels in *S. miltiorrhiza* leaves.

### *SmMYB1* promotes phenolic acid accumulation and activates the *CYP98A14* promoter

To investigate the role of *SmMYB1* in phenolic acid biosynthesis, we selected three *SmMYB1*-overexpressing (*SmMYB1-OE*) hairy root lines and three SRDX repression lines (*SmMYB1-SRDX*) for further analysis (Supplementary Fig. S7). *SmMYB1* was expressed at significantly higher levels in all of these lines than in the control (Supplementary Fig. S8).

**Fig. 4:**
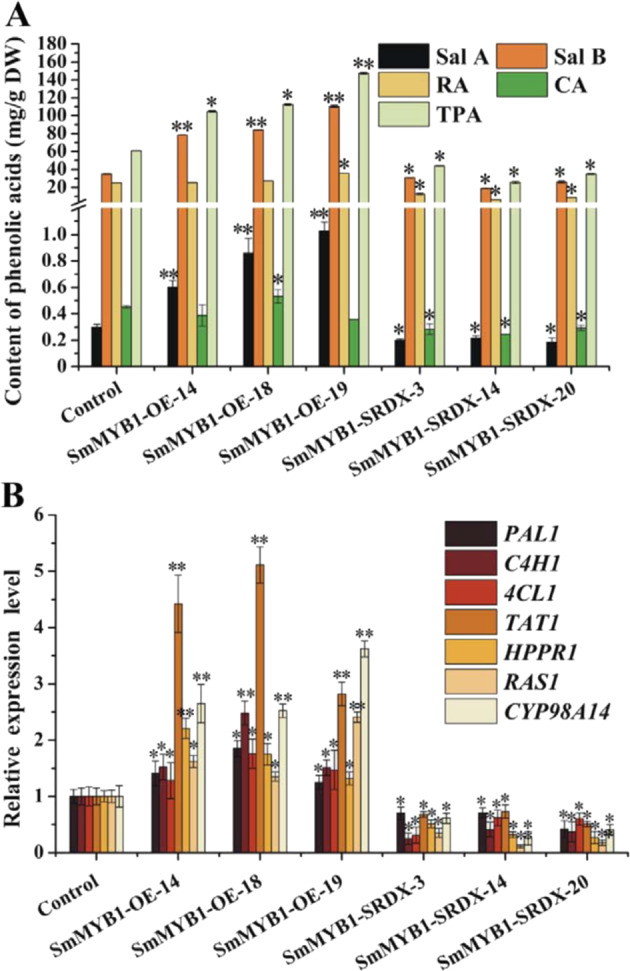
Expression of key genes and phenolic acid contents in transgenic *SmMYB1* hairy roots. **A** Phenolic acid (salvianolic acid A, Sal A; salvianolic acid B, Sal B; rosmarinic acid, RA; caffeic acid, CA; total phenolic acids, TPA) contents in *SmMYB1-OE* and *SmMYB1-SRDX* lines, as measured by HPLC. **B** Expression of genes involved in phenolic acid biosynthesis in *SmMYB1-OE* (overexpression) and *SmMYB1-SRDX* (repression) lines. The fold changes of the relative gene expression level in transgenic hairy roots were normalized to those in the control. The error bars indicate the standard errors of the means. The asterisks on the bars indicate significant differences compared with the control according to *t-*tests at two different significance levels (**P < 0.01; *P < 0.05). The experiment was replicated three times

We performed HPLC to measure phenolic acid yields from the transgenic hairy root lines. The average phenolic acid yield in the *SmMYB1-OE* lines was 121.64 mg/g dry weight (DW) compared to only 60.84 mg/g DW in the control. By contrast, the average phenolic acid yield in the *SmMYB1-SRDX* lines was 34.91 mg/g DW (Fig. 4A). In addition, the expression of the phenolic acid biosynthesis-related genes *PAL1*, *C4H1*, *4CL1*, *TAT1*, *HPPR1*, *RAS1*, and *CYP98A14* was upregulated in the *SmMYB1-*overexpression lines but strongly downregulated in the *SmMYB1-SRDX* lines compared to the control. The expression of *TAT1* and *CYP98A14* was most significantly affected in these lines (Fig. 4B). Taken together, these results indicate that *SmMYB1* positively regulates phenolic acid levels in *S. miltiorrhiza*.

**Fig. 5:**
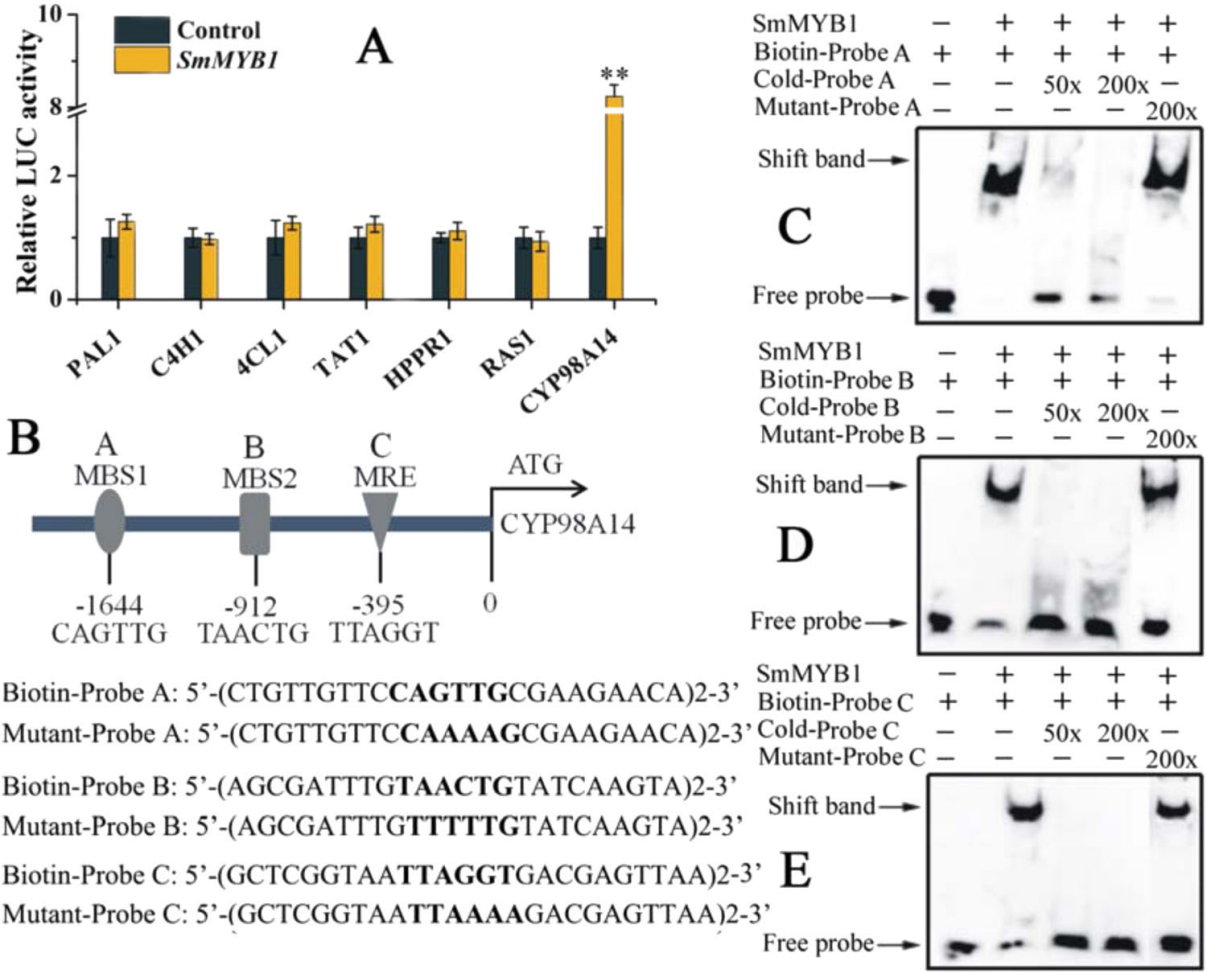
Validation of the targeted genes bound by SmMYB1. **A** Dual-LUC assay results. Shown is the effector construct that contained *SmMYB1* driven by the CaMV 35S promoter. The promoters of candidate genes (*PAL1*, *C4H1*, *4CL1*, *TAT1*, *HPPR1*, *RAS1*, and *CYP98A14*) fused to *LUC* were used as reporter constructs. An effector vector not carrying *SmMYB1* served as a negative control. Fold changes in the relative LUC activity were normalized to the control activity. The error bars indicate the standard errors of the means. The asterisks indicate significant differences according to *t*-tests at two different significance levels (**P < 0.01; *P < 0.05). The experiment was replicated three times. **B** MYB-binding elements in *CYP98A14* and the probe sequences for EMSA analysis. **C**–**E** EMSA of SmMYB1. The Cold-Probe sequences were the same as the labeled probes but without biotin labels

The promoters of all seven of these phenolic acid biosynthesis pathway genes contain MYB-binding elements (Supplementary Fig. S9). We, therefore, performed dual-LUC assays to determine whether SmMYB1 activates the promoters of these genes in vivo. Among these genes, a significantly higher LUC/REN ratio was detected only when *pGreen 0800::CYP98A14* constructs WERE USED, with the ratio increasing more than 7-fold in the presence of SmMYB1 compared to that of the control (Fig. 5A). We searched for *cis*-elements that bind to MYB proteins within the promoter region of *CYP98A14* using the PlantCARE program (http://bioinformatics.psb.ugent.be/beg/tools/plantcare). Three MYB-binding *cis*-elements were found: the CAGTTG (MBS1), TAACTG (MBS2), and TTAGGT (MRE) motifs (Fig. 5B). To further verify the binding of SmMYB1 to the MYB-binding *cis*-elements in the *CYP98A14* promoter, we performed EMSAs, the results of which confirmed that SmMYB1 binds to the promoter region of *CYP98A14* (Fig. 5C–E). These findings indicate that SmMYB1 activates *CYP98A14* expression both in vivo and in vitro, possibly through direct binding of the *cis*-element in the promoter of *CYP98A14*.

### SmMYB1 regulates anthocyanin accumulation by affecting *CHI* and *ANS* expression

**Fig. 6:**
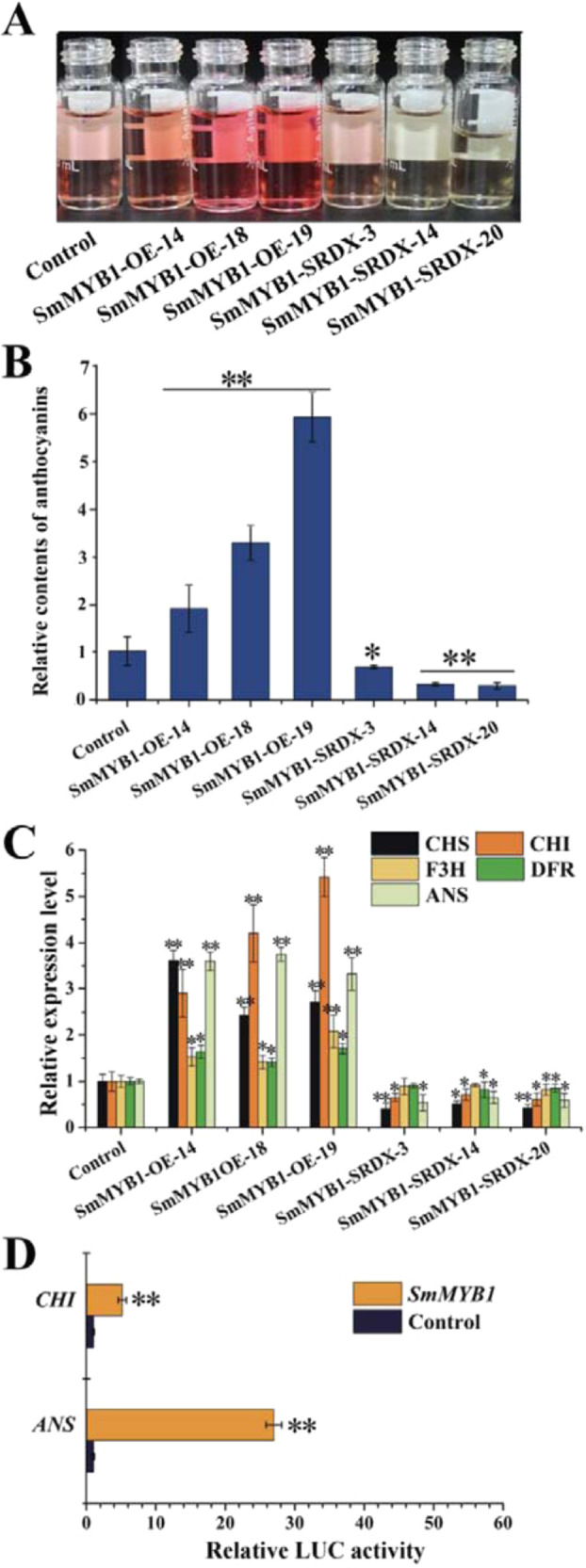
Role of *SmMYB1* in promoting anthocyanin accumulation in *S. miltiorrhiza* transgenic hairy root lines. **A**, **B** Anthocyanin contents and the appearance of extracts from transgenic hairy root lines. Fold changes of the relative anthocyanin contents in transgenic hairy roots were normalized to the contents in the control. Control: empty vector; SmMYB-OE: *SmMYB* overexpression; SmMYB-SRDX: *SmMYB-SRDX* repression. **A** Expression profiles of genes involved in anthocyanin biosynthesis. Fold changes of the relative gene expression level in transgenic hairy roots were normalized to the level in the control. **B** Activation of *CHI* and *ANS* expression, as revealed by dual-LUC assays. The effector construct contained *SmMYB1* driven by the CaMV 35S promoter. The promoters of candidate genes (*CHI*, *ANS*) fused to *LUC* were used as reporter constructs. An effector vector not carrying *SmMYB1* served as a negative control. The error bars indicate the standard errors of the means. The asterisks indicate significant differences according to *t*-tests at two different significance levels (**P < 0.01; *P < 0.05). The experiment was replicated three times

Phenylalanine is a common precursor for phenolic acid and anthocyanin biosynthesis (Fig. 1). Therefore, we measured anthocyanin accumulation in the transgenic hairy root lines. Anthocyanin levels were significantly higher in the *SmMYB1-OE* lines than in the control and drastically lower in the *SmMYB1-SRDX* lines (Fig. 6A, B). Furthermore, the expression of anthocyanin biosynthesis pathway genes *CHS*, *CHI*, *F3H*, *ANS*, and *DFR* was significantly upregulated in the *SmMYB1-OE* lines compared with that in the control, whereas that of *CHS*, *CHI*, *ANS*, and *DFR* were significantly downregulated in the *SmMYB1-SRDX* lines (Fig. 6C).

Since overexpressing *SmMYB1* significantly increased the expression of *CHI* and *ANS*, we searched for MYB-binding elements in the promoters of these genes. We then performed a dual-LUC assay to determine whether SmMYB1 activates the transcription of *CHI* and *ANS* in vivo. The LUC/REN ratio using *CHI* and *ANS* increased 5.16-fold and 26.98-fold, respectively, in the presence of SmMYB1 compared to that of the control (Fig. 6D). These findings indicate that SmMYB1 increases *CHI* and *ANS* expression, thereby promoting anthocyanin biosynthesis. Furthermore, overexpressing *SmMYB1* not only increased phenolic acid accumulation but also increased anthocyanin biosynthesis in the *S. miltiorrhiza* hairy root lines.

### Validation of the protein-protein interaction between SmMYB1 and SmMYC2

**Fig. 7:**
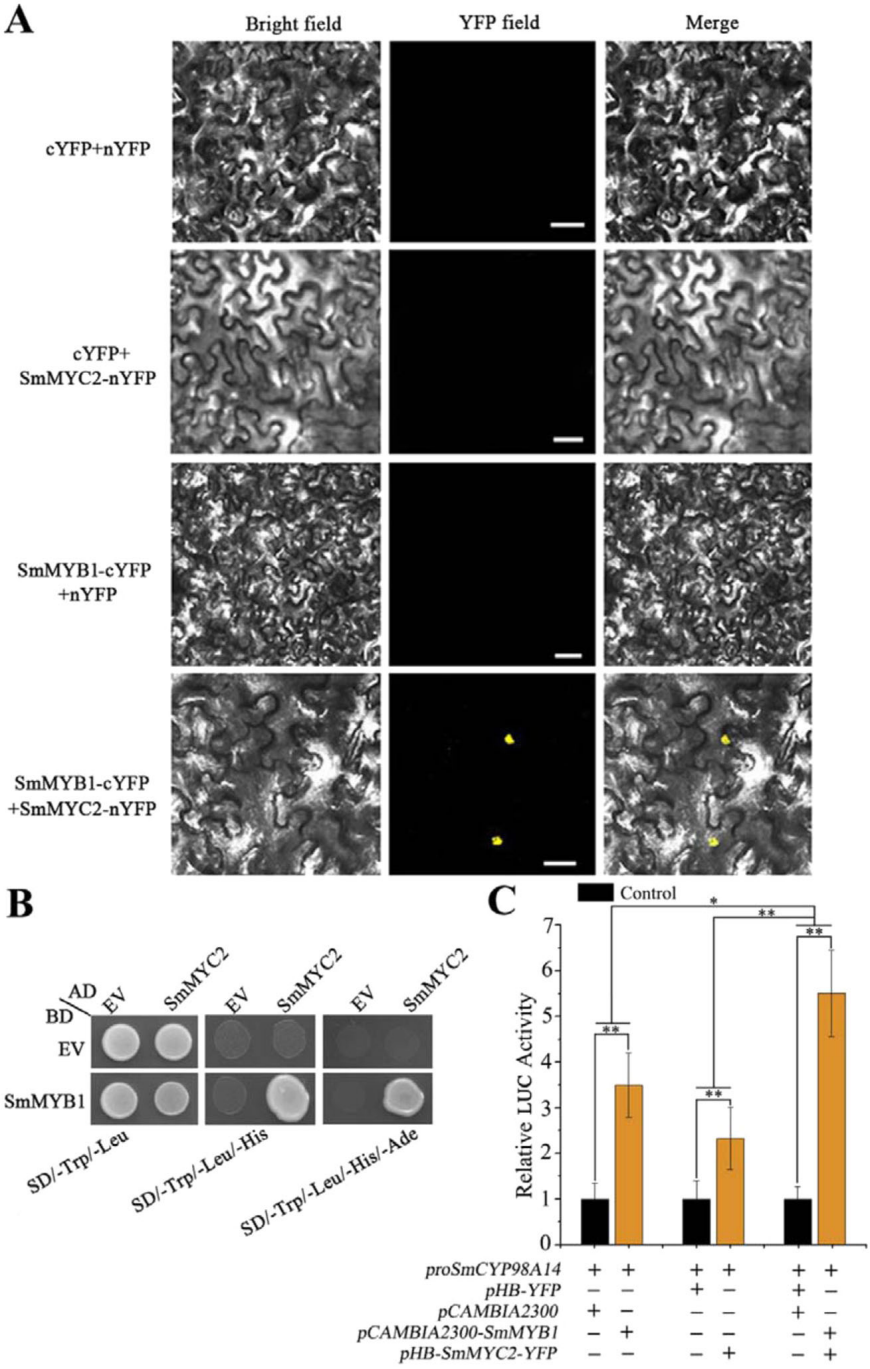
SmMYB1 interacts with SmMYC2 to promote *CYP98A14* expression. **A** BiFC assay; scale bars, 50 µm. **B** Y2H assay. AD: pGADT7 vector; BD: pGBKT7 vector. **C** Coexpressing *SmMYB1* and *SmMYC2* enhanced the activation of the reporter construct, as revealed by dual-LUC assays. The effector constructs contained *SmMYB1* or *SmMYC2* driven by the CaMV 35S promoter. *proCYP98A14::LUC* was used as the reporter construct. Effector vectors not carrying *SmMYB1* or *SmMYC2* were used as negative controls. Fold changes of the relative LUC activity were normalized to the activity of the control. The asterisks indicate significant differences according to *t*-tests at two different significance levels (**P < 0.01; *P < 0.05). The experiment was replicated three times

MYC2 is a central transcriptional regulator of jasmonate signaling^41^. Analysis of a previously generated transcriptome dataset^19^ indicated that *SmMYC2* is strongly induced by MeJA. Therefore, we performed BiFC and Y2H assays to investigate the interaction between SmMYB1 and SmMYC2 (GenBank accession number MN400428). In the BiFC assay, the polypeptides cYFP and nYFP were able to bind together only when fusion constructs expressing *SmMYB1-cYFP* and *nYFP-SmMYC2* were paired together. Specifically, yellow fluorescence was observed only when *N. benthamiana* leaves expressing the resulting full-length *YFP* were exposed to blue light, whereas leaves harboring the control constructs did not fluoresce under blue light (Fig. 7A). In the Y2H assay, yeast cells harboring the recombinant plasmids *SmMYC2-AD* and *SmMYB1-BD* grew normally on SD media lacking the nutrients l-tryptophan, l-leucine, l-histidine HCl monohydrate, and adenine hemisulfate (SD/−Trp/−Leu/−His/−Ade) (Fig. 7B).

### SmMYB1/SmMYC2 additively activates the *CYP98A14* promoter

Overexpressing *SmMYC2* increased phenolic acid production in *S. miltiorrhiza* by activating the expression of *CYP98A14*^42,43^. Thus, the finding that SmMYC2 has the same target as SmMYB1 prompted us to examine whether SmMYC2 and SmMYB1 together would activate *CYP98A14* expression to a greater extent than either protein would separately. We used dual-LUC assays to assess the promoter activity of *CYP98A14* with the effectors SmMYB1 and SmMYC2 delivered individually or in combination into *N. benthamiana* leaf cells. As expected, the activation of the *CYP98A14* promoter was significantly enhanced by coexpressing SmMYC2 and SmMYB1 compared to that as a result of either protein alone (Fig. 7C). Taken together, these results suggest that SmMYB1 increases the transcriptional activation activity of *CYP98A14* by possibly interacting with SmMYC2 to additively promote phenolic acid biosynthesis.

## Discussion

### *SmMYB1* might serve as an additional link between MeJA induction and phenolic acid biosynthesis

Both biotic and abiotic elicitors, such as YE, Ag^+^, Co^2+^, α-amino isobutyric acid, β-aminobutyric acid (BABA), MeJA, GA_3_, Eth, SA, and wounding, increase the accumulation of secondary metabolites in plants, and in turn, the following processes have been shown to be affected: the control of tanshinone and phenolic acid biosynthesis in *S. miltiorrhiza*^43–46^; anthocyanin, lignin and suberin biosynthesis in *Arabidopsis*^47–49^; stilbene biosynthesis in *Vitis vinifera*^50^; and proanthocyanidin and flavonol biosynthesis in apple^51^. MeJA can be used to simulate natural wounding. In *Arabidopsis*, cell wall damage induces lignin biosynthesis, which is regulated by a reactive oxygen species- and jasmonic acid-dependent process^47^. Whether phenolic acid accumulation can be induced by wounding through *SmMYB1* under jasmonic acid-dependent processes in *S. miltiorrhiza* needs to be further studied.

MYB TFs play key roles in regulating secondary metabolite biosynthesis in plants. Although various phenolic acid biosynthesis-related genes have been isolated and characterized, we need to identify a few R2R3-MYB TFs that function as positive regulators in promoting phenolic acid accumulation; in this regard, it is valuable to elucidate the molecular mechanism through which phenolic acid biosynthesis in *S. miltiorrhiza* is regulated. In the current study, the R2R3-MYB TF-encoding gene *SmMYB1* was chosen for further analysis based on transcriptional data mining, because *SmMYB1* (SmiContig1043) was strongly responsive to MeJA induction at the two MeJA induction points and exhibited an expression profile similar to that of biosynthesis pathway genes (*TAT1*, *PAL1*, *HPPR1*, *RAS1*, and *CYP98A14*) in our transcriptome dataset. In addition, *SmMYB1* exhibited expression patterns similar to the pattern exhibited by key phenolic acid biosynthesis pathway genes, such as *RAS1* and *CYP98A14* (Fig. S6).

*S. miltiorrhiza* contains 110 R2R3-MYB family members, as revealed by genomic analysis^28^. Based on the conserved motifs in their amino acid sequences, these R2R3-MYBs could be further divided into 37 subgroups in *S. miltiorrhiza*^28,31^. Predictions made by comparing R2R3-MYBs in *S. miltiorrhiza* with those in other plant species suggested that S3, S4, S5, S6, S7, S13, and S21 subgroup members are involved in phenolic acid biosynthesis, while S4 and S20 subgroup members regulate terpenoid biosynthesis^28,31^. SmMYB1 is closely evolutionarily related to subgroup five proteins (Fig. 2A), suggesting that it might regulate phenolic acid biosynthesis^32,52^. Here, we isolated and identified the novel R2R3-MYB TF *SmMYB1* from *S. miltiorrhiza*. The average total phenolic acid content was 2-fold greater in the *SmMYB1-OE* hairy root lines than in the control but only 57% of the control amount in the *SmMYB1-SRDX* lines (Fig. 4A). These results indicate that *SmMYB1* positively regulates MeJA-mediated phenolic acid biosynthesis in *S. miltiorrhiza*.

In fact, except for *SmMYB1*, another MYB TF (whose unigene ID is SmiContig1859), which shows the greatest upregulation under MeJA induction in our transcriptome data set, was identified recently, designated as *SmMYB2*. SmMYB2 cannot interact with SmMYC2 like SmMYB1 but significantly increases the accumulation of salvianolic acid by activating the same target *CYP98A14* gene as SmMYB1 does in overexpression transgenic hairy roots of *S. miltiorrhiza*^45^. In addition to *SmMYB2*, *SmMYB1* is obviously another important R2R3-MYB member identified by us that serves as a link between MeJA induction and phenolic acid biosynthesis in *S. miltiorrhiza*.

### SmMYB1 positively regulates phenolic acid biosynthesis in *S. miltiorrhiza* by activating *CYP98A14*

In the current study, overexpressing *SmMYB1* not only upregulated the expression of its target gene *CYP98A14* but also upregulated the expression of six genes in the phenolic acid biosynthesis pathway. Overexpressing *SmERF1L1* promotes the expression of *DXS2*, *DXR*, *HMGS*, *CPS1*, and *KSL1*, but only *DXR* is a target of SmERF1L1^12^. In *SmERF128-*overexpressing transgenic hairy roots, the expression of *CYP76AH1*, *KSL1*, *CPS1*, *HDS*, *MDS*, and *DXR* was activated, but only *CYP76AH1*, *KSL1*, and *CPS1* were confirmed to be targets of SmERF128^53^. Based on these findings, it appears that the ectopic expression of certain TF-encoding genes upregulates the expression of not only their target genes but also other genes in the same biosynthesis pathway.

Although several MYB TFs, such as *MYB36* and *MYB39*, have been functionally identified in *S. miltiorrhiza*^31,32^, direct evidence for the regulation of their target genes in vivo and in vitro is lacking. In the current study, we isolated and functionally identified the R2R3-MYB TF *SmMYB1*, which upregulated the expression of genes (including *PAL1*, *C4H1*, *4CL1*, *TAT1*, *HPPR1*, *CYP98A14*, and *RAS1*) in the phenolic acid biosynthesis pathway in *SmMYB1*-overexpressing lines and consequently increased their phenolic acid contents. We verified that SmMYB1 activated *CYP98A14* expression via dual-LUC assays and EMSA (Fig. 5A–E). Whether SmMYB1 can directly activate *CYP98A14* expression needs to be further validated by ChIP-qPCR experiments.

### *SmMYB1* positively regulates anthocyanin accumulation in *S. miltiorrhiza*

We also investigated anthocyanin accumulation and the expression of anthocyanin biosynthesis-related genes in *S. miltiorrhiza*. Interestingly, overexpressing *SmMYB1* upregulated the expression of anthocyanin biosynthesis-related genes such as *CHI* and *ANS*, consequently increasing anthocyanin contents. Dual-LUC assays further confirmed that SmMYB1 promotes anthocyanin biosynthesis, most likely by activating *CHI* and *ANS*. However, *CHS*, *CHI*, *F3H*, and *FLS* are the targets of the R2R3-MYB TFs MYB11, MYB12, and MYB111 in *A. thaliana*^54^. VvMYB14 and VvMYB15 specifically activate the promoter of the *STS* gene and regulate stilbene biosynthesis in grapevine^50^, and MsMYB22 activates flavonol pathways by binding to the *FLS* gene promoter in apple^51^. *ODO1*, a member of the R2R3-type MYB family, regulates the biosynthesis of fragrance-related volatiles by activating the promoter of the 5-enol-pyruvylshikimate-3-phosphate synthase (EPSPS) gene in the shikimate biosynthesis pathway in petunia flowers^55^. AtMYB12 was shown to target the *ENO* and *DAHPS* genes in the shikimate pathway and *PAL1*, *CHS*, and *F3H* in the phenylpropanoid pathway in *A. thaliana*, which not only increases the supply of carbon from primary metabolism but also fuels the shikimate and phenylalanine biosynthesis pathways to supply more aromatic amino acids for secondary metabolism^54^. Therefore, different R2R3-MYB TFs function in different regulatory pathways in different plant species.

The R2R3-MYB TF *SmMYB39* negatively regulates phenolic acid accumulation in Danshen^32^, while overexpressing *SmMYB111* and *SmMYB2* promotes phenolic acid accumulation in *S. miltiorrhiza* hairy root lines^23,42,45^. Because only a few R2R3-MYB TFs with confirmed roles in promoting phenolic acid accumulation have been identified, the molecular mechanism through which phenolic acid biosynthesis is regulated in *S. miltiorrhiza* remains unknown. Taken together, our results provide new insights into the regulatory mechanism utilized by the R2R3-MYB TFs *SmMYC2* and *SmMYB1*, which not only positively regulate phenolic acid biosynthesis but also promote anthocyanin accumulation in *S. miltiorrhiza* hairy root lines.

### SmMYC2 interacts with SmMYB1 to additively activate the expression of *CYP98A14*

MYC2 is a central transcriptional regulator of jasmonate signaling^41^. *SmMYC2* activates the expression of *CYP98A14* to promote phenolic acid accumulation in *S. miltiorrhiza*^42,43,56^. To date, the molecular mechanism through which SmMYC2 coregulates phenolic acid biosynthesis together with MYB proteins in *S. miltiorrhiza* remains unknown. Previous studies demonstrated that various R2R3-MYB TFs do not act alone but instead interact with other proteins, such as bHLH and WD40 TFs, to form transcriptional complexes that regulate flavonoid biosynthesis^51,54^. In the present study, we demonstrated that SmMYC2 interacts with SmMYB1 to additively activate the expression of *CYP98A14*. The APETALA2/ethylene-responsive factor (AP2/ERF) TFs SmERF1L1 and SmERF128 positively regulate diterpenoid tanshinone biosynthesis by activating the expression of genes involved in the tanshinone biosynthesis pathway in *S. miltiorrhiza*^12,53^. It would be interesting to determine whether AP2/ERF TFs interact or cooperate with MYB TFs or MYC2 to promote phenolic acid or anthocyanin accumulation in *S. miltiorrhiza*.

### A proposed model for the role of *SmMYB1* in phenolic acid biosynthesis

**Fig. 8:**
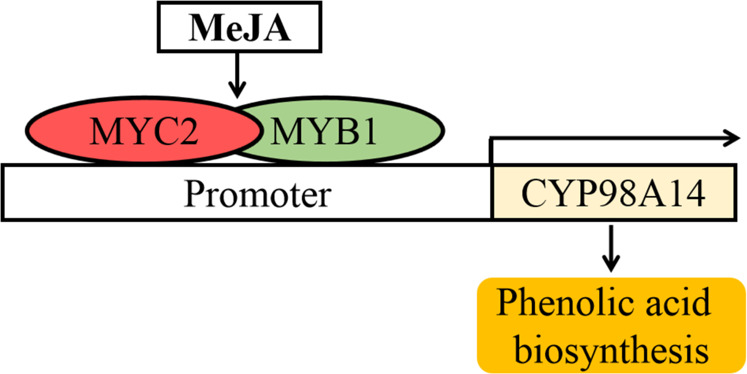
Proposed model for the role of *SmMYB1* in modulating phenolic acid biosynthesis in *S. miltiorrhiza*. As TFs in response to MeJA, MYC2 and MYB1 can interact to additively promote *CYP98A14* expression, thereby promoting the accumulation of phenolic acids

In *S. miltiorrhiza*, overexpression of *SmMYC2* increases the production of phenolic acids in transgenic hairy roots^42^. Based on the present findings and those of previous studies in *A. thaliana* and other plant species^57–59^, we propose a model describing the role of *SmMYB1* in modulating phenolic acid biosynthesis in *S. miltiorrhiza*. According to this model, *SmMYB1* is induced by MeJA signals and activates the expression of *CYP98A14* by binding to *cis*-elements (MBS1, MBS2, MRE) in its promoter, thereby promoting phenolic acid accumulation. As a positive regulator of MeJA signaling, SmMYC2 possibly interacts with SmMYB1 to additively activate the expression of *CYP98A14*, which jointly promotes phenolic acid accumulation in *S. miltiorrhiza* (Fig. 8).

## Supplementary information

Supplementary Figures and Legends

## References

[1] Xu H (2010). Metabolic regulation and genetic engineering of pharmaceutical component tanshinone biosynthesis in *Salvia miltiorrhiza*. J. Med. Plants Res..

[2] Shi, M., Liao, P., Nile, S., Georgiev, M. & Kai, G. Biotechnological exploration of transformed root culture for value-added products. *Trends Biotechnol*. 10.1016/j.tibtech.2020.06.012 (2020) 10.1016/j.tibtech.2020.06.012 32690221

[3] Zhou L, Zuo Z, Chow M (2005). Danshen: An overview of its chemistry pharmacology pharmacokinetics and clinical use. J. Clin. Pharmacol..

[4] Shi M (2016). Enhanced diterpene tanshinone accumulation and bioactivity of transgenic *Salvia miltiorrhiza* hairy roots by pathway engineering. J. Agric. Food Chem..

[5] Zhou W (2016). Molecular cloning and characterization of two 1-deoxy-d-xylulose-5-phosphate synthase genes involved in tanshionone biosynthesis in *Salvia miltiorrhiza*. Mol. Breed..

[6] Ren J, Fu L, Nile SH, Zhang J, Kai G (2019). *Salvia miltiorrhiza* in treating cardiovascular diseases: a review on its pharmacological and clinical applications. Front. Pharmacol..

[7] Shi M, Huang F, Deng C, Wang Y, Kai G (2019). Bioactivities biosynthesis and biotechnological production of phenolic acids in *Salvia miltiorrhiza*. Crit. Rev. Food Sci..

[8] Liao P (2009). Molecular cloning characterization and expression analysis of a new gene encoding 3-hydroxy-3-methylglutaryl coenzyme A reductase from *Salvia miltiorrhiza*. Acta Physiol. Plant.

[9] Deng C (2020). ABA-responsive transcription factor bZIP1 is involved in modulating biosynthesis of phenolic acids and tanshinones in *Salvia miltiorrhiza*. J. Exp. Bot..

[10] Kai G (2011). Metabolic engineering tanshinone biosynthetic pathway in *Salvia miltiorrhiza* hairy root cultures. Metab. Eng..

[11] Guo J (2013). CYP76AH1 catalyzes turnover of miltiradiene in tanshinones biosynthesis and enables heterologous production of ferruginol in yeasts. PNAS.

[12] Huang Q (2019). The AP2/ERF transcription factor SmERF1L1 regulates the biosynthesis of tanshinones and phenolic acids in *Salvia miltiorrhiza*. Food Chem..

[13] Zhang Y (2014). Pathway engineering for phenolic acid accumulations in *Salvia miltiorrhiza* by combinational genetic manipulation. Metab. Eng..

[14] Sun M (2019). The biosynthesis of phenolic acids is positively regulated by the JA-responsive transcription factor ERF115 in *Salvia miltiorrhiza*. J. Exp. Bot..

[15] Di P (2013). ¹³C tracer reveals phenolic acids biosynthesis in hairy root cultures of *Salvia miltiorrhiza*. ACS Chem. Biol..

[16] Zhao S, Hu Z, Liu D, Leung F (2006). Two divergent members of 4-coumarate: coenzyme A ligase from *Salvia miltiorrhiza Bunge*: cDNA cloning and functional study. J. Integr. Plant Biol..

[17] Huang B (2008). Characterization and expression profiling of tyrosine aminotransferase gene from *Salvia miltiorrhiza* (Dan-shen) in rosmarinic acid biosynthesis pathway. Mol. Biol. Rep..

[18] Xiao Y (2009). Methyl jasmonate dramatically enhances the accumulation of phenolic acids in *Salvia miltiorrhiza* hairy root cultures. Physiol. Plant.

[19] Zhou W (2017). Comprehensive transcriptome profiling of *Salvia miltiorrhiza* for discovery of genes associated with the biosynthesis of tanshinone and salvianolic acids. Sci. Rep..

[20] Winkel-Shirley B (2001). Flavonoid biosynthesis: a colorful model for genetics biochemistry cell biology and biotechnology. Plant Physiol..

[21] Porth I, Hamberger B, White R, Ritland K (2011). Defense mechanisms against herbivory in *Picea*: sequence evolution and expression regulation of gene family members in the phenylpropanoid pathway. BMC Genomics.

[22] Deng C (2019). Tanshinone production could be increased by the expression of SmWRKY2 in *Salvia miltiorrhiza* hairy roots. Plant Sci..

[23] Li S (2018). SmMYB111 is a key factor to phenolic acid biosynthesis and interacts with both SmTTG1 and SmbHLH51 in *Salvia miltiorrhiza*. J. Agric. Food Chem..

[24] Cao W (2018). Transcription factor SmWRKY1 positively promotes the biosynthesis of tanshinones in *Salvia miltiorrhiza*. Front. Plant Sci..

[25] Liu Y, Patra B, Pattanaik S, Wang Y, Yuan L (2019). GATA and phytochrome interacting factor transcription factors regulate light-induced vindoline biosynthesis in *Catharanthus roseus*. Plant Physiol..

[26] Hiratsu K, Matsui K, Koyama T, Ohme-Takagi M (2003). Dominant repression of target genes by chimeric repressors that include the EAR motif, a repression domain, in *Arabidopsis*. Plant J..

[27] Dubos C (2010). MYB transcription factors in *Arabidopsis*. Trends Plant Sci..

[28] Li C, Lu S (2014). Genome-wide characterization and comparative analysis of R2R3-MYB transcription factors shows the complexity of MYB-associated regulatory networks in *Salvia miltiorrhiza*. BMC Genomics.

[29] Huang W (2013). A R2R3-MYB transcription factor from *Epimedium sagittatum* regulates the flavonoid biosynthetic pathway. PLos ONE.

[30] Vimolmangkang S, Han Y, Wei G, Korban S (2013). An apple MYB transcription factor, MdMYB3, is involved in regulation of anthocyanin biosynthesis and flower development. BMC Plant Biol..

[31] Ding K (2017). SmMYB36 a novel R2R3-MYB transcription factor enhances tanshinone accumulation and decreases phenolic acid content in *Salvia miltiorrhiza* hairy roots. Sci. Rep..

[32] Zhang S (2013). Cloning and characterization of a putative R2R3 MYB transcriptional repressor of the rosmarinic acid biosynthetic pathway from *Salvia miltiorrhiza*. PLos ONE.

[33] Kai G (2010). Characterization expression profiling and functional identification of a gene encoding geranylgeranyl diphosphate synthase from *Salvia miltiorrhiza*. Biotechnol. Bioproc. E..

[34] Zhang L (2011). Molecular cloning and expression analysis of a new putative gene encoding 3-hydroxy-3-methylglutaryl-CoA synthase from *Salvia miltiorrhiza*. Acta Physiol. Plant.

[35] Zhou W (2016). Mapping of Ppd-B1 a major candidate gene for late heading on wild emmer chromosome arm 2BS and assessment of its interactions with early heading QTLs on 3AL. PLoS ONE.

[36] Thompson JD, Gibson TJ, Plewniak F, Jeanmougin F, Higgins DG (1997). The CLUSTAL X windows interface flexible strategies for multiple sequence alignment aided by quality analysis tools. Nucleic Acids Res..

[37] Tamura K, Stecher G, Peterson D, Filipski A, Kumar S (2013). MEGA6: molecular evolutionary genetics analysis version 6.0. Mol. Biol. Evol..

[38] Shi M (2016). Methyl jasmonate induction of tanshinone biosynthesis in *Salvia miltiorrhiza* hairy roots is mediated by JASMONATE ZIM-DOMAIN repressor proteins. Sci. Rep..

[39] Zhou W, Gong Y, Huang C, Gao F (2012). Molecular cloning and function analysis of flavonoid 3’-hydroxylase gene in the purple-fleshed sweet potato (*Ipomoea batatas*). Mol. Biol. Rep..

[40] Lv Z (2016). Overexpression of a novel NAC domain-containing transcription factor gene (AaNAC1) enhances the content of artemisinin and increases tolerance to drought and botrytis cinerea in A*rtemisia annua*. Plant Cell Physiol..

[41] Sasaki-Sekimoto Y, Saito H, Masuda S, Shirasu K, Ohta H (2014). Comprehensive analysis of protein interactions between JAZ proteins and bHLH transcription factors that negatively regulate jasmonate signaling. Plant Signal. Behav..

[42] Yang N (2017). Overexpression of SmMYC2 increases the production of phenolic acids in *Salvia miltiorrhiza*. Front. Plant Sci..

[43] Zhou Y (2016). SmMYC2a and SmMYC2b played similar but irreplaceable roles in regulating the biosynthesis of tanshinones and phenolic acids in *Salvia miltiorrhiza*. Sci. Rep..

[44] Ge X, Wu J (2005). Tanshinone production and isoprenoid pathways in *Salvia miltiorrhiza* hairy roots induced by Ag+ and yeast elicitor. Plant Sci..

[45] Deng C (2020). SmMYB2 promotes salvianolic acid biosynthesis in the medicinal herb *Salvia miltiorrhiza*. J. Integr. Plant Biol..

[46] Hao X (2015). Effects of methyl jasmonate and salicylic acid on the tanshinone production and biosynthetic genes expression in transgenic *Salvia miltiorrhiza* hairy roots. Biotechnol. Appl Biochem..

[47] Denness L (2011). Cell wall damage-induced lignin biosynthesis is regulated by a reactive oxygen species- and jasmonic acid-dependent process in *Arabidopsis*. Plant Physiol..

[48] Stracke R (2007). Differential regulation of closely related R2R3-MYB transcription factors controls flavonol accumulation in different parts of the *Arabidopsis thaliana* seedling. Plant J..

[49] Gou M (2017). The MYB107 transcription factor positively regulates biosynthesis. Plant Physiol..

[50] Höll J (2013). The R2R3-MYB transcription factors MYB14 and MYB15 regulate stilbene biosynthesis in *Vitis vinifera*. Plant Cell.

[51] Wang N (2017). MYB12 and MYB22 play essential roles in proanthocyanidin and flavonol synthesis in red-fleshed apple (*Malus sieversii f. niedzwetzkyana*). Plant J..

[52] Zhang J (2017). Overexpression of SmMYB9b enhances tanshinone concentration in *Salvia miltiorrhiza* hairy roots. Plant Cell Rep..

[53] Zhang Y, Ji A, Xu Z, Luo H, Song J (2019). The AP2/ERF transcription factor SmERF128 positively regulates diterpenoid biosynthesis in *Salvia miltiorrhiza*. Plant Mol. Biol..

[54] Zhang Y (2015). Multi-level engineering facilitates the production of phenylpropanoid compounds in tomato. Nat. Commun..

[55] Verdonk J (2005). ODORANT1 regulates fragrance biosynthesis in petunia flowers. Plant Cell.

[56] Du T (2018). SmbHLH37 Functions antagonistically with SmMYC2 in regulating jasmonate-mediated biosynthesis of phenolic acids in *Salvia miltiorrhiza*. Front. Plant Sci..

[57] Nesi N, Jond C, Debeaujon I, Caboche M, Lepiniec L (2001). The *Arabidopsis* TT2 gene encodes an R2R3 MYB domain protein that acts as a key determinant for proanthocyanidin accumulation in developing seed. Plant Cell.

[58] Chini A, Boter M, Solano R (2009). Plant oxylipins: COI1/JAZs/MYC2 as the core jasmonic acid-signalling module. FEBS J..

[59] Zhao S, Zhang J, Tan R, Yang L, Zheng X (2015). Enhancing diterpenoid concentration in *Salvia miltiorrhiza* hairy roots through pathway engineering with maize C1 transcription factor. J. Exp. Bot..

