# Supplementary material for: The methyl jasmonate-responsive transcription factor SmMYB1 promotes phenolic acid biosynthesis in Salvia miltiorrhiza
Source: Hortic Res. 2021 Jan 1;8:10. doi: 10.1038/s41438-020-00443-5 (PMC7775463; doi:10.1038/s41438-020-00443-5)
Supplement: Supplementary file 1 — Supplementary Figures and Legends [file 41438_2020_443_MOESM1_ESM.doc]

**
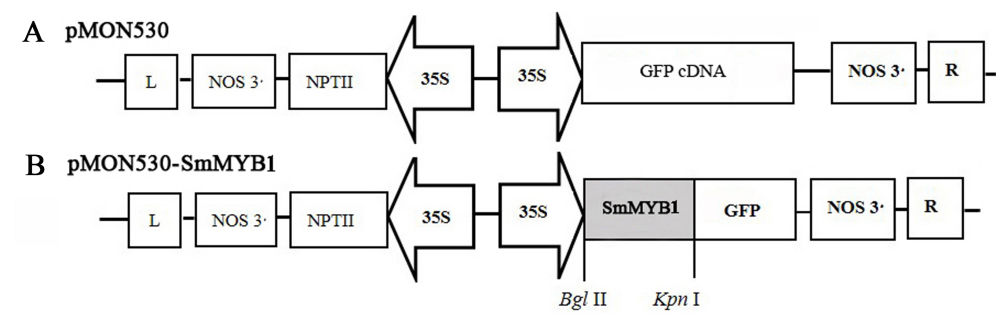
**

**Fig. S1.** Schematic representation of the pMON530-SmMYB1 constructs.

(A) pMON530-GFP.

1. pMON530-SmMYB1-GFP.


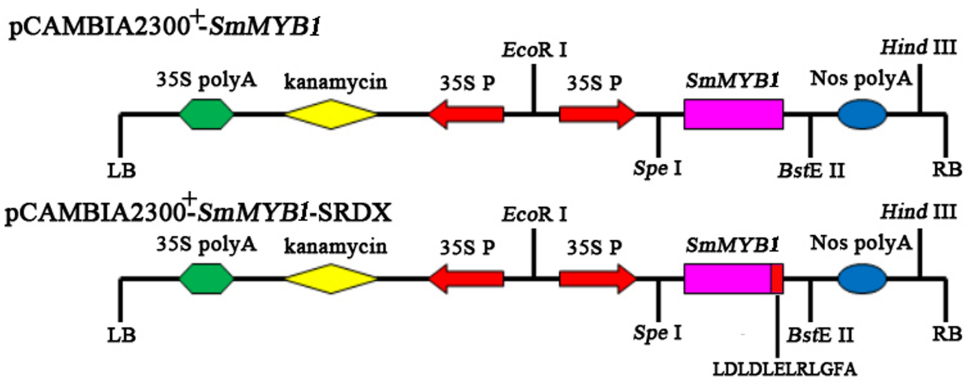


**Fig. S2.** Schematic representation of the pCAMBIA2300-SmMYB1 and pCAMBIA2300-SmMYB1-SRDX constructs.


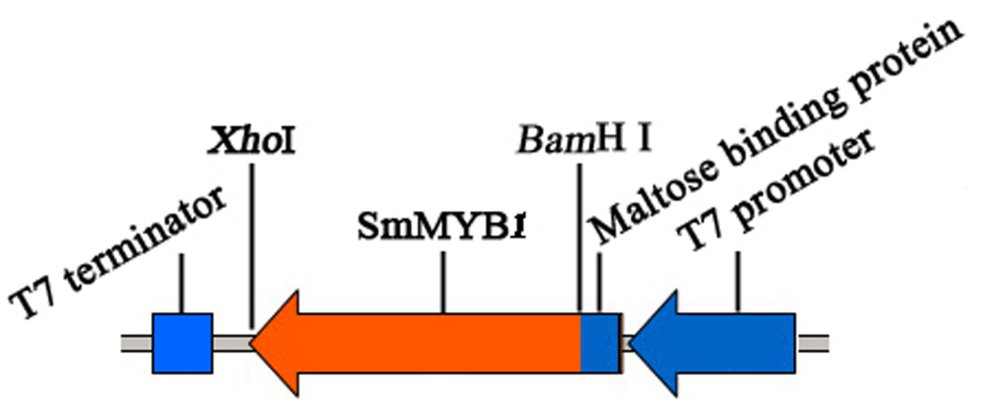


**Fig. S3.** Schematic representation of the pETMAL_C_-H construct.


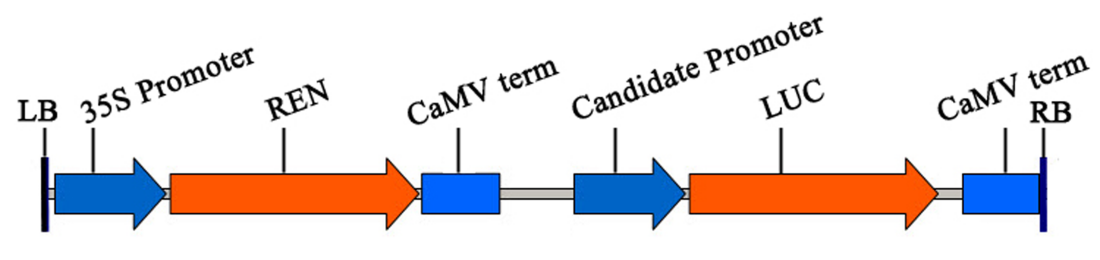


**Fig. S4.** Schematic representation of the pGreen II 0800-LUC construct.


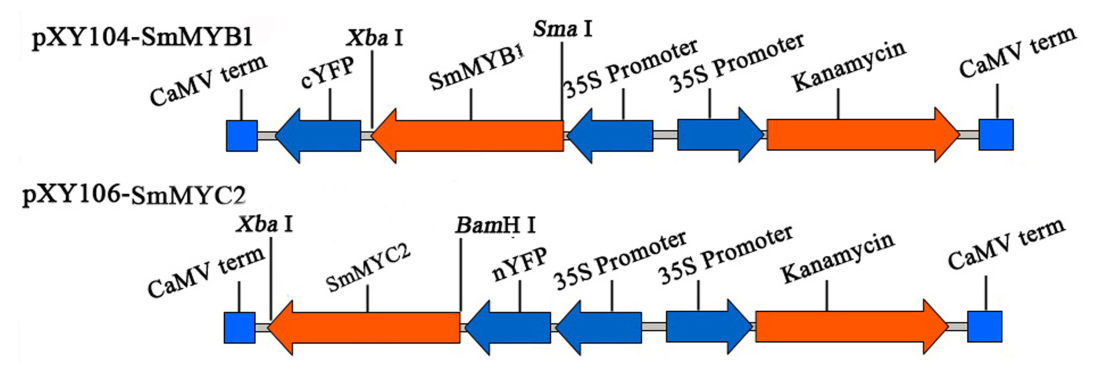


**Fig. S5.** Schematic representation of the pXY104 and pXY106 constructs.


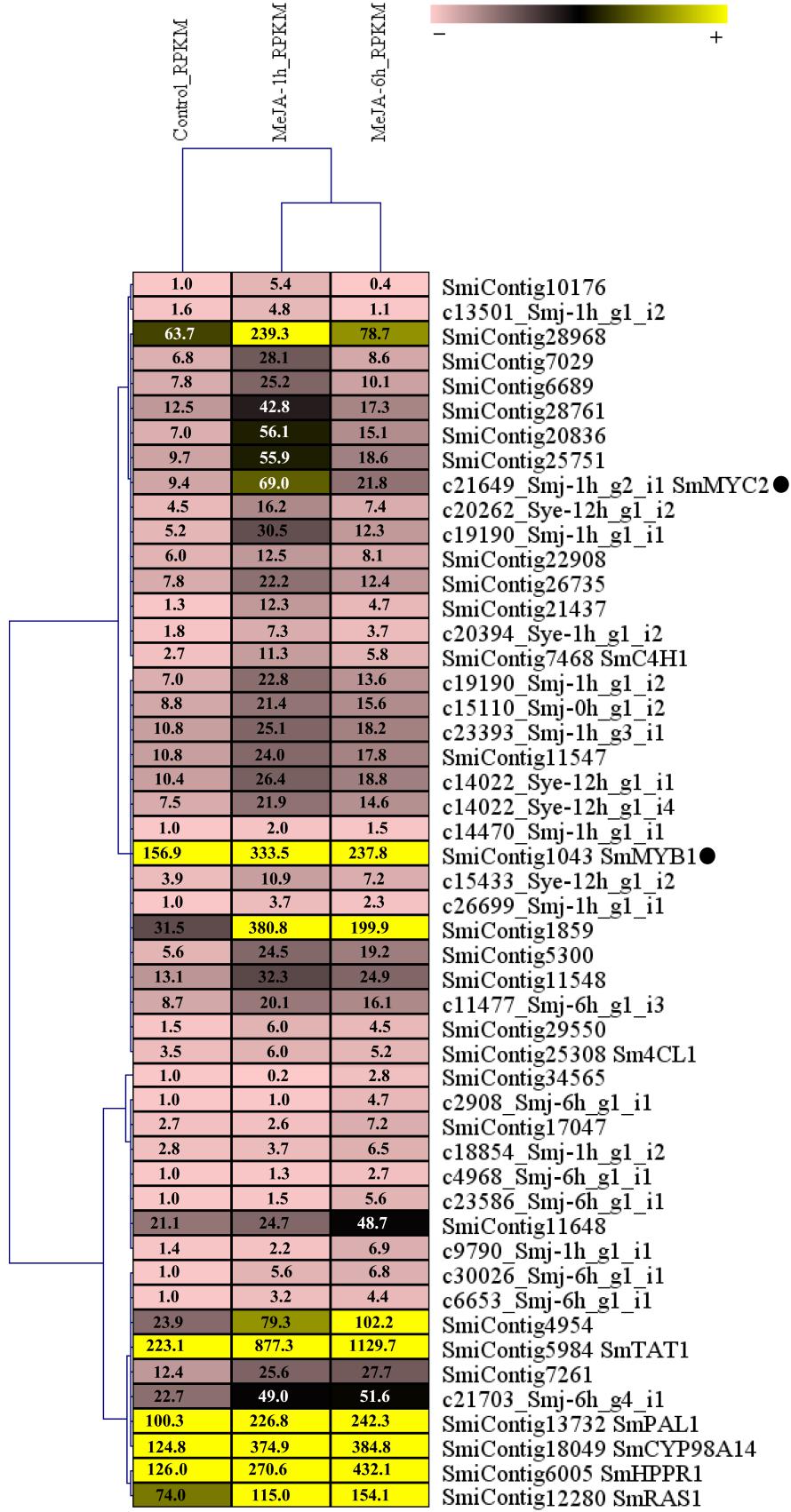


**Fig. S6.** Hierarchical cluster analysis of MYB TFs.

Hierarchical cluster analysis of MYB TFs responding to MeJA induction. The RPKM values in the color key from light red to yellow denote the expression levels of the candidate unigenes from low to high value. Numbers in the color box denotes the RPKM value of candidate unigenes.


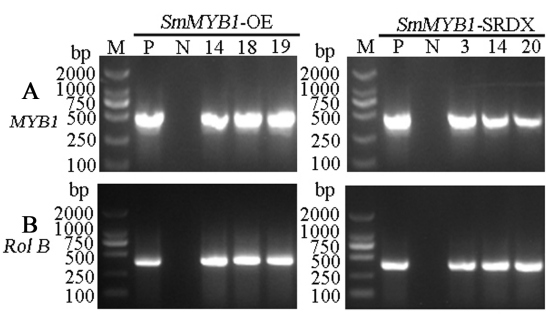


**Fig. S7.** Identification of transgenic hairy root lines by PCR.


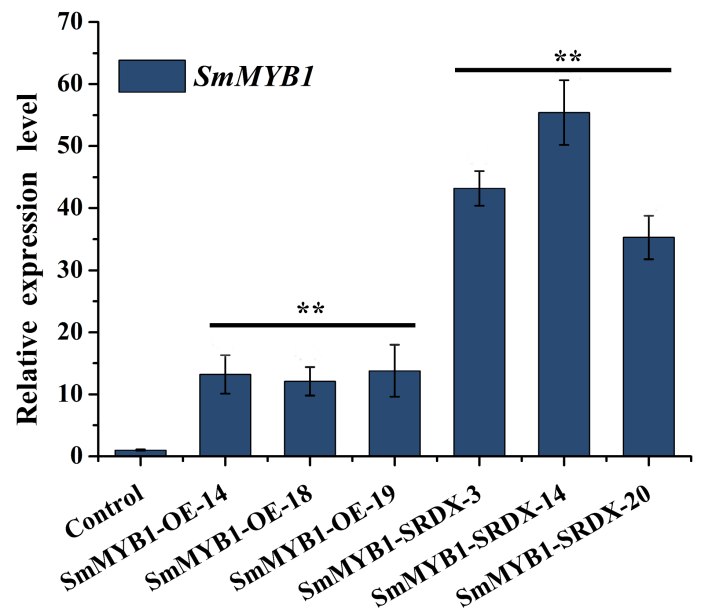


**Fig. S8.** Expression profiles of *SmMYB1* in overexpression (*SmMYB1-OE*) and suppression (*SmMYB1-SRDX*) hairy root lines, as determined by qRT-PCR.


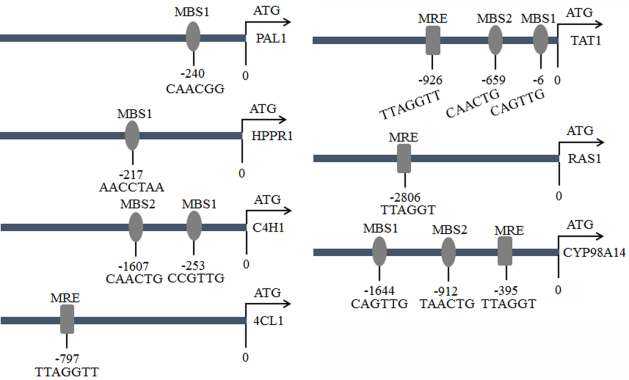


**Fig. S9.** MYB binding elements and sites in the promoter regions of phenolic acid biosynthetic genes.

**Table S1** Primer sequences used in this study.

| **Primers** | **Sequences (5’-3’)** | **Notes** |
| --- | --- | --- |
| PAL1-1065F | TACCTCGTCGCCCTATGCCAAG | Used in qRT-PCR analysis |
| PAL1-1182R | ATTGACGCCCATTGTGAGAGTT |  |
| HPPR1-614F | TGACTCCAGAAACAACCCACATT |  |
| HPPR1-733R | CCCAGACGACCCTCCACAAG |  |
| C4L1-264F | ATTCGCATTCGCATTTCTCGG |  |
| C4L1-405R | GCGGCGTAGTGCTTCACCTTT |  |
| TAT1-227F | CAACTGCTGGTCTTCCACAAAC |  |
| TAT1-369R | GCGAGCCAAAACGGACA |  |
| C4H1-1021F | CCAGGAGTCCAAATAACAGAGC |  |
| C4H1-1183R | GCCACCAAGCGTTCACCAAGAT |  |
| RAS1-648F | CGAGATCGCCTACTCCAAGTTCAAG |  |
| RAS1-865R | AGATGGCGTTACCGAAGTATCCCTG |  |
| CYP98A14-375F | GGTCTGTACCGTCGTCCTCTTCTCC |  |
| CYP98A14-544R | ACAAGGCTGGTATTTGGGAAAAGGT |  |
| AACT1-285F | AGTCTGTGCCTCTGGAATGAAAGC |  |
| AACT1-479R | ACATCCCAGAGTCCGTCCTTCAG |  |
| KSL1- 1480F | GTGTGACCCTTCTGCTAGCA |  |
| KSL1-1630R | TGCATTGTCTTGGGAAGATG |  |
| GGPPS1-603F | GCTGTGCTCGCAGGGGATG |  |
| GGPPS1-774R | ATCGCCGGTGCAGTTCAGG |  |
| DXS2-1828F | TTGGAGATTGGGAAGGGAAGGAT |  |
| DXS2-1980R | AGGCTTGCAGAATCTCGCATCAG |  |
| HMGR-982F | TCGTTTTCAATAAGTCGAGTAGA |  |
| HMGR-1142R | ATTCTGAAGGAAGTCCAAAACAT |  |
| HMGS-282F | GATGAGCATGACAGCGGTTACTTC |  |
| HMGS-362R | GGATTTGCTCTTGTCGAGTACGGT |  |
| DXR-1248F | CGACGAGAAAATCGGATACCTGG |  |
| DXR-1424R | CATACAAGAGCAGGACTCAAACCG |  |
| CPS1-F214 | ACTACCGTTCATCAAGGCCA |  |
| CPS1-R421 | CCTCGAGTTGATTCTGCACG |  |
| CYP76AH1-F221 | TCCATCTCGGCAGCCTCTACAC |  |
| CYP76AH1-R304 | GAGAAGACCTGCCCGTGCCT |  |
| Actin F | AGCACCGAGCAGCATGAAGATT |  |
| Actin R | AGCAAAGCAGCGAACGAAGAGT |  |
| SmMYC2 ORFF | ATGATTGATTACCGCACGCCGA | Used in gene amplication |
| SmMYC2 ORFR | CTATCTAATCTCAGCAACTTTAGATATCAA |  |
| SmMYB1 ORFF | ATGGGAAGATCCGCTTGCTG |  |
| SmMYB1 ORFR | TTAAAGAAGCTGCATATACTCGGC |  |
| SmMYB1F | AAACCCATGAGAGTCTCCTCGC | Used in qRT-PCR analysis |
| SmMYB1R | TTAAAGAAGCTGCATATACTCGGC |  |
| Biotin-Probe A CYP98A14-F1 | (CTGTTGTTC**CAGTTG**CGAAGAACA)_2_ | Used in EMSA analysis |
| Biotin-Probe A CYP98A14-R1 | (TGTTCTTCG**CAACTG**GAACAACAG)_2_ |  |
| Mutant-Probe A CYP98A14-F1 | (CTGTTGTTC**CAAAAG**CGAAGAACA)_2_ |  |
| Mutant-Probe A CYP98A14-R1 | (TGTTCTTCG**CTTTTG**GAACAACAG)_2_ |  |
| Biotin-Probe B CYP98A14-F2 | (AGCGATTTG**TAACTG**TATCAAGTA)_2_ |  |
| Biotin-Probe B CYP98A14-R2 | (TACTTGATA**CAGTTA**CAAATCGCT)_2_ |  |
| Mutant-Probe B CYP98A14-F2 | (AGCGATTTG**TTTTTG**TATCAAGTA)_2_ |  |
| Mutant-Probe B CYP98A14-R2 | (TACTTGATA**CAAAAA**CAAATCGCT)_2_ |  |
| Biotin-Probe C CYP98A14-F3  Biotin-Probe C CYP98A14-R3 | (GCTCGGTAA**TTAGGT**GACGAGTTAA)_2_  (TTAACTCGTC**ACCTAA**TTACCGAGC)_2_ |  |
| Mutant-Probe C CYP98A14-F3 | (GCTCGGTAA**TTAAAA**GACGAGTTAA)_2_ |  |
| Mutant-Probe C CYP98A14-R3 | (TTAACTCGTC**TTTTAA**TTACCGAGC)_2_ |  |
| Promoter-PAL1-F | TTTCTAGGAGGGAGTGACACGGGTT | Used in promoters amplication |
| Promoter-PAL1-R | GGCTGCTGCGGATGAGCTTGCCTCAC |  |
| Promoter-HPPR1-F | GTTGTATTTTGGGTGGCTGCTATCGTTG |  |
| Promoter-HPPR1-R | ATTTTCGCGGCAGCGGCGGCGTTGGT |  |
| Promoter-C4H1-F | CAGCTACAGGTCACGGACGAGAAAGGTTA |  |
| Promoter-C4H1-R | AAAATGGTTTGCGGTCTGCAGTGGCGGTGGT |  |
| Promoter-4CL1-F | ACCGACAACAAAAAGCCGCCAGTAAC |  |
| Promoter-4CL1-R | TTCTTCACTTTCTCTAACTAATGTTTTCTG |  |
| Promoter-TAT1-F | AACTGGCTAATGTGGCTAATAAATGC |  |
| Promoter-TAT1-R | TGCCGCAACTGAATCGAGAGATCTGA |  |
| Promoter-RAS1-F | GTTTAACCATCAGGTCCATATTCCGCAA |  |
| Promoter-RAS1-R | TTTGAGAGAGACAGAGAGAGGGGGAAGAAT |  |
| Promoter-CYP98A14-F | TCCCCCACCCCCAAAACAATCCTT |  |
| Promoter-CYP98A14-R | GGCTGCTGCGGATGAGCTTGCCTCACT |  |
| SmMYB1- NOS-F | TCCCAAGATATGAAAAATAGCAAAG | Used in transgenic examination |
| NOS-R | CCCGATCTAGTAACATAGATGACA |  |
| RolB-F | GCTCTTGCAGTGCTAGATTT |  |
| RolB-R | GAAGGTGCAAGCTACCTCTC |  |

**Note:** The response element for MYB transcription factor is indicated by underline, and the homologous mutant element is indicated by black frame.
